# How general is the natural frequency effect? The case of joint probabilities

**DOI:** 10.3389/fpsyg.2024.1296359

**Published:** 2024-04-10

**Authors:** Nathalie Stegmüller, Karin Binder, Stefan Krauss

**Affiliations:** ^1^Mathematics Education, Faculty of Mathematics, University of Regensburg, Regensburg, Germany; ^2^Mathematics Education, Institute of Mathematics, Ludwig Maximilian University Munich, Munich, Germany

**Keywords:** joint probabilities, Bayesian reasoning, natural frequencies, visualization, net diagram

## Abstract

Natural frequencies are known to improve performance in Bayesian reasoning. However, their impact in situations with two binary events has not yet been completely examined, as most researchers in the last 30 years focused only on conditional probabilities. Nevertheless, situations with two binary events consist of 16 elementary probabilities and so we widen the scope and focus on joint probabilities. In this article, we theoretically elaborate on the importance of joint probabilities, for example, in situations like the Linda problem. Furthermore, we implemented a study in a 2×5×2 design with the factors information format (probabilities vs. natural frequencies), visualization type (“Bayesian text” vs. tree diagram vs. double tree diagram vs. net diagram vs. 2×2 table), and context (mammography vs. economics problem). Additionally, all four “joint questions” (i.e., $P(A\cap B),P(\bar{A}\cap B),P(\bar{A}\cap \bar{B}),P(A\cap \bar{B}))$ were asked for. The main factor of interest was whether there is a format effect in the five visualization types named above. Surprisingly, the advantage of natural frequencies was not found for joint probabilities and, most strikingly, the format interacted with the visualization type. Specifically, while people’s understanding of joint probabilities in a double tree seems to be worse than the understanding of the corresponding natural frequencies (and, thus, the frequency effect holds true), the opposite seems to be true in the 2 × 2 table. Hence, the advantage of natural frequencies compared to probabilities in typical Bayesian tasks cannot be found in the same way when joint probability or frequency tasks are asked.

## 1 Introduction

There is an interesting tension in empirical research on the understanding of *joint probabilities* (formal: e.g., P(A∩B)). On one hand, researchers have stressed the importance of comprehending joint probabilities, e.g., in the legal context (O’Grady, 2023) and conducted empirical studies (e.g., Tversky and Kahneman, 1974; Donati et al., 2019). On the other hand, psychological studies mostly just ask for a *qualitative comparison* of P(A) and P(A∩B) without the need for participants to assess a concrete joint probability. Let us, for example, consider the most famous instance of the so-called conjunction fallacy, namely the Linda problem (introduced by Tversky and Kahneman, 1983).

*Linda is 31 years old, single, outspoken, and very bright. She majored in philosophy. As a student, she was deeply concerned with issues of discrimination and social justice, and she also participated in anti-nuclear demonstrations. Which is more probable?*

1. *Linda is a bank teller.*
2. *Linda is a bank teller and is active in the feminist movement.*

Let “A” be the event “being active in the feminist movement” and “B” “being a bank teller.” Since B∩A (being a bank teller *and* being active in the feminist movement) is a subset of B (being a bank teller), the single event B is more probable than both events at the same time. Formally, the multiplication rule concerning joint probabilities is P(B∩A) = P(B) ⋅ P(A|B) and because P(B) must be multiplied with a probability, i.e., a number between 0 and 1, P(B∩A) cannot be larger than P(B).

Yet, the fact that no concrete probability has to be estimated or calculated stands in strong contrast to the way *conditional probabilities* are examined in cognitive psychology, for example, in the framework of *Bayesian reasoning* in which specific estimates have to be given by participants (see Theoretical Framework).

For requesting a concrete joint probability in the Linda task, participants, for instance, might be asked:

*Linda is 31 years old, single, outspoken, and very bright. She majored in philosophy. As a student, she was deeply concerned with issues of discrimination and social justice, and she also participated in anti-nuclear demonstrations. Assume that the probability that Linda is a bank teller is 5%. Assume that the probability that she is active in the feminist movement, if she is a bank teller, is 20%. What is the probability that she is a bank teller and active in the feminist movement?*

Now, the *multiplication rule* based on the given information yields P(B∩A) = P(B) ⋅ P(A|B) = 5% ⋅ 20% = 1%. Considering this rule, it becomes clear that joint probabilities, i.e., P(A∩B), are deeply interwoven with conditional probabilities, i.e., P(A|B). Joint probabilities are even used for defining conditional probabilities in mathematics (P(A|B) = P(A∩B)/P(B)). The tension in psychological research is that joint probabilities are stressed as very relevant, but at the same time concrete joint probabilities usually do not have to be calculated by participants. In the present study, we investigate people’s assessment of concrete numerical values of joint probabilities. The main aim is to explore, whether the so-called “natural frequency effect” (that helps participants assess conditional probabilities) can also be found for joint probability judgments.

## 2 Theoretical framework

In the following, we first embed the structure of the Linda problem in the larger framework of Bayesian reasoning situations consisting of two binary events. In general, in the statistical world of two binary events A and B (with the counter events $\bar{A}$ and $\bar{B}$), one can consider 16 different elementary probabilities:^1^

- Four *marginal probabilities*: $P(A),P(\bar{A}),P(B),P(\bar{B})$
- Four *joint probabilities*: $P(A\cap B),P(\bar{A}\cap B),P(\bar{A}\cap \bar{B}),P(A\cap \bar{B})$
- Eight *conditional probabilities*:

$\begin{array}{l} P(A|B),P(A|\bar{B}),P(\bar{A}|B),P(\bar{A}|\bar{B}),P(B|A),P(B|\bar{A}),P(\bar{B}|A),\\ P(\bar{B}|\bar{A}) \end{array}$

Note that in the case of stochastic independence of both events, P(A|B) equals P(A) and, thus, the multiplication rule can be simplified:

- A and B are stochastic dependent: P(B∩A) = P(B) ⋅ P(A|B)
- A and B are stochastic independent: P(B∩A) = P(B) ⋅ P(A)

Ignoring the dependency of two events was, by the way, one of several problems in the famous miscarriage of justice concerning Sally Clark (Colmez and Schneps, 2013) or the one of Kathleen Folbigg (O’Grady, 2023), which again stresses the importance of understanding joint probabilities (including concrete values). After two infants of Sally Clark died shortly after birth, she was convicted of murdering her children. The court knew that the sudden infant death syndrome (SIDS) occurs with a chance of about 1 in 8500 cases. After not only one infant but two of her children died, it was considered to be very unlikely that this happened by chance, particularly under the wrong assumption that these two deaths were *independent* of each other. Consequently, the chance for two children suffering from SIDS was calculated as $\frac{1}{8500}$ ⋅ $\frac{1}{8500}$ (≈0.0000014%), whereupon she was convicted of being a murderer. However, a second SIDS is more probable given a first one already happened (Glinge et al., 2023). As soon as this was stated clearly, Clark was released from prison (after three years of her sentence); nevertheless, her life had been destroyed (Colmez and Schneps, 2013). In a similar, more recent criminal case, Kathleen Folbigg was convicted of murdering three of her infant children and of manslaughter of her fourth child (Phillips, 2022). This verdict was based on the same misunderstanding as Clark’s—the court assumed that four children could not independently die by accident but only by being murdered. After scientists, though, had analyzed the case for about 20 years and had proven a gene mutation in the family, Folbigg was finally released from prison in 2023 (Wells et al., 2023).

### 2.1 Bayesian reasoning and natural frequencies

**Figure 1:**
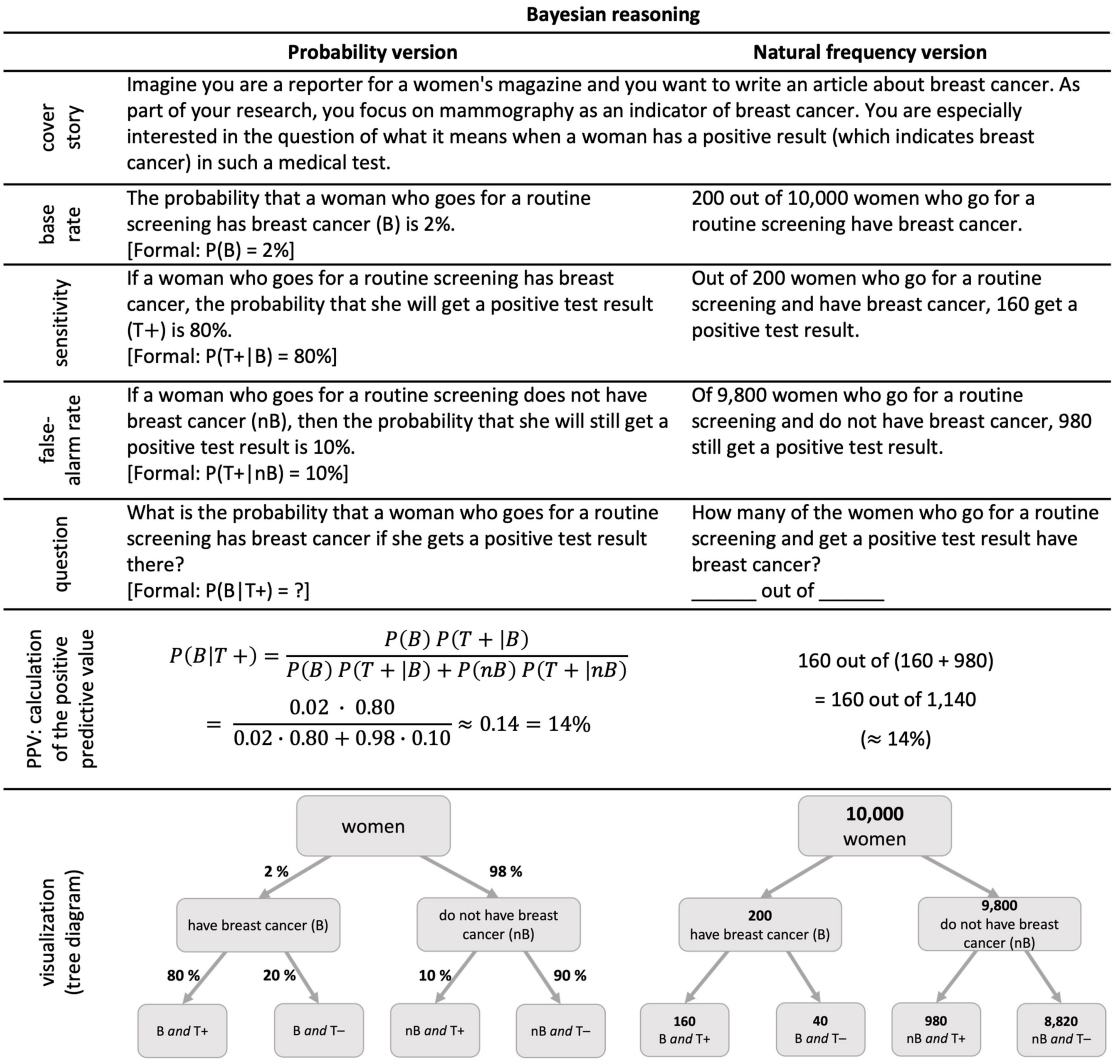
The famous mammography task (adapted from Eddy, 1982): probability version (left) and natural frequency version (right).

In psychological research on situations with two binary events, typically *Bayesian reasoning* is investigated empirically. For this, a specific set of probabilities is given, and a concrete probability is required (Figure 1). In more detail, the “positive predictive value” P(B|T+) has to be inferred from (1) the base rate P(B), (2) the sensitivity P(T + |B), and (3) the false-alarm rate P(T + |nB), which reflects the typical setting of diagnostic situations. Figure 1 displays the famous mammography task (adapted from Eddy, 1982). Since the issue of joint probabilities is strongly related to such diagnostic reasoning, we first take a short look at the research area of Bayesian reasoning. Many studies documented the difficulties people—laymen and experts like physicians—have with such problems, especially when they are formulated in terms of probabilities (Figure 1, left; Gigerenzer and Hoffrage, 1995; Garcia-Retamero and Hoffrage, 2013; Binder et al., 2015; Bruckmaier et al., 2019).

In research on Bayesian reasoning, it turned out that a reformulation with so-called “natural frequencies” (Figure 1, right side) helps people to understand such situations (Gigerenzer and Hoffrage, 1995; Siegrist and Keller, 2011). Natural frequencies are a pair of natural numbers a and b (a ≤ b), which are equivalent to percentages and used as “a out of b” (Krauss et al., 2020). Sometimes, people distinguish between “percentages” and “natural frequencies” instead of “probabilities” and “natural frequencies” (e.g., Knapp et al., 2009). In this article, we use the latter distinction. A meta-analysis revealed that on average in probability versions (without visualization) usually only 4% of people can solve such tasks correctly, while, in natural frequency versions (also without visualizations), 24% of people find the correct solution (McDowell and Jacobs, 2017).

Natural frequencies are helpful because the calculations are simpler compared to the probability version (Figure 1) and, thus, the solution can be accessed more easily (Gigerenzer and Hoffrage, 1995). The higher solution rates can, therefore, also be explained by the number of mental steps that are needed to solve the problem. In the probability format, the correct solution has to be calculated using a sophisticated formula, while people only have to identify two correct numbers and do a simple addition in the frequency format. Studies show that Bayesian tasks are solved more correctly the less mental steps are needed (Ayal and Beyth-Marom, 2014).

Note that, in the tree diagram (Figure 1, left), conditional probabilities are depicted at the lower arrows, for instance the sensitivity P(T + |B) of 80%, represented at the very left branch. Joint probabilities are *not* depicted. However, P(B∩T+), for example, might be calculated according to the multiplication rule above by P(B∩T+) = P(B) ⋅ P(T + |B) = 2% ⋅ 80% = 1.6%.

In typical Bayesian reasoning tasks, joint probabilities are neither given nor asked for. For an exception for *giving* joint probabilities see the “short menu” in Gigerenzer and Hoffrage (1995); for exceptions for *asking* for joint probabilities see Böcherer-Linder and Eichler (2017), Bruckmaier et al. (2019), or Binder et al. (2020).

From the perspective of the widespread research on Bayesian reasoning and the largely documented effect of natural frequencies, however, it is an interesting question, whether natural frequency formulations would also help understanding notorious joint probabilities. This is especially intriguing since Bayes formula (Figure 1, left) could alternatively be written as


$$P\left(B|T+\right)=\frac{P\left(B\cap T+\right)}{P\left(B\cap T+\right)+P\left(nB\cap T+\right)}$$

While 16 probabilities are available in statistical situations with two binary events, empirical research has, to a very large extent, primarily focused on Bayesian reasoning tasks. The enormous effect of natural frequencies in such basic diagnostic tasks motivates the question what happens in related or extended problem-solving situations.

### 2.2 Extensions of Bayesian reasoning—and the respective help of natural frequencies

Before we address a possible generalization of the natural frequency effect from Bayesian reasoning to joint probabilities in detail (see section 2.3), we first shed light on the potential of natural frequencies in alternative extensions of Bayesian reasoning. The following paragraphs summarize various possible extensions of Bayesian reasoning and whether studies document that natural frequencies also help in these cases. Interestingly, there seems to be a clear format effect as long as conditional probabilities are considered. When it comes to joint probabilities, though, there does not seem to be an overall format effect in favor of natural frequencies because the evidence is mixed.

To explain extensions 1–3, medical contexts are used in the following.

#### 2.2.1 Increasing the number of tests (extension 1a)

One possible extension of Bayesian reasoning would be to vary the number of medical tests applied. In the context of breast cancer, for instance, after a mammography screening, an ultrasound test might be applied to verify the test results (which would yield another level in the tree diagram in Figure 1, e.g., Binder et al., 2018). Krauss et al. (1999), for example, found that natural frequency versions were more than four times as likely to be solved correctly than probability versions. Similar results can be found in Woike et al. (2017).

#### 2.2.2 Increasing the number of test (or criterion) values (extension 1b)

Another way of altering Bayesian reasoning is to increase the number of test and/or criterion values. For instance, a medical test might have three different outcomes (positive, negative, unclear). In the same manner, a medical test can be sensitive to two different diseases, which would result in three possible criterion values (e.g., diabetes type 1, diabetes type 2, or healthy). Modeling three (or even more) possible test outcomes as well as three (or even more) possible health statuses would lead to three (or more) nodes in a tree diagram in the second or in the third level, respectively. Formulating tasks in such complex situations in natural frequencies leads to about 50% of correct performances of participants (Hoffrage et al., 2015). Binder and Krauss (under review) confirm these results and give an extensive overview of studies on such types of generalization (i.e., 1a and 1b).

#### 2.2.3 Covariational reasoning (extension 2)

Another interesting way of extending the classical Bayesian reasoning task would be to consider whether people are aware of the consequences of *changing* one of the three input variables (i.e., base rate, sensitivity, false-alarm rate) on the positive predictive value. Even though such kind of reasoning is very complex, some people, nevertheless, can correctly judge the direction of change of the positive predictive value after a respective training, when it is based on the natural frequency concept (Steib et al., 2023; Büchter et al., 2024).

#### 2.2.4 Communication skills (extension 3)

The *communication quality* in Bayesian situations is a further aspect worth to consider. Since Bayesian situations often occur in medical contexts in which a physician is supposed to advise patients, the way of (verbally) communicating the meaning of a positive test result is very important (Gigerenzer et al., 1998; Brose et al., 2023). Unfortunately, counselors are not always communicating the results in a correct and comprehensible way (Gigerenzer et al., 1998; Ellis and Brase, 2015; Prinz et al., 2015) and medical students cannot even identify a high-quality communication with the correct value when it is presented as one out of several short video clips (Böcherer-Linder et al., 2022). To improve (pictorial) communication, the Harding Center for Risk Literacy developed fact boxes and icon boxes (Schwartz et al., 2007; McDowell et al., 2019), which are also based on the concept of natural frequencies. Clearly, verbal and pictorial communication can benefit from the frequency effect.

### 2.3 The issue of joint probabilities: Do natural frequencies help?

The extensions discussed so far (1–3) deal with *conditional probabilities*. However, there are 16 elementary probabilities available in Bayesian situations (see above). Thus, it is an interesting question whether natural frequencies help in a similar way when questions on *joint probabilities* are posed. In the following paragraphs, we analyze empirical evidence collected so far. First (in 2.3.1), we summarize experimental results concerning the *qualitative* comparison of P(A∩B) and P(A). Afterwards (in 2.3.2), we turn to *quantitative* tasks in which a *concrete* probability is asked for. Finally, we conclude that the evidence regarding the help of natural frequencies concerning joint probabilities is mixed and explain the limitations of the studies conducted so far.

#### 2.3.1 Qualitative comparison of P(A∩B) and P(A)

Besides the original study of the Linda problem by Tversky and Kahneman (1983), many studies document that people consider the second option with two events at the same time as more likely as the first option with only one event (e.g., Charness et al., 2009; Donati et al., 2019). However, as demonstrated above, one single event is *always* more probable than the simultaneous occurrence of this event *and* an additional event.

Since the background information on Linda, which is irrelevant for the multiplication rule, seems to make option 2 more plausible, Tversky and Kahneman (1983) explain people’s difficulties by the *representativeness heuristic*, which can sometimes lead to misjudgments. Yet, there are alternative explanations, for instance, that the word “and” in everyday communication has many different meanings (Mellers et al., 2001; Hertwig et al., 2008). Another explanation of the fallacy is that people interpret the first event “Linda is a bank teller” in reminiscence to the second option as “Linda is a bank teller and is NOT active in the feminist movement” (Hertwig et al., 2008).

Nonetheless, similar difficulties occur in related tasks like for example in “rolling the dice” (Tversky and Kahneman, 1983) in which the events are not formulated literally, and, therefore, such linguistic problems cannot explain participants’ misconceptions.

*Consider a regular six-sided dice with four green faces and two red faces. The dice will be rolled 20 times and the sequence of greens (G) and reds (R) will be recorded. You are asked to select one sequence from a set of three and you will win $25 if the sequence you chose appears on successive rolls of the dice. Please check the sequence of greens and reds on which you prefer to bet.*

1. *RGRRR*
2. *GRGRRR*
3. *GRRRRR*

In this task, three options (instead of two) are given, but, again, one (1.) is a subset of another (2.). Most participants orientated themselves on the probabilities of rolling a green face (4/6) and of rolling a red face (2/6) and, therefore, chose sequence 2, which includes more green faces compared to sequence 1, both absolutely and relatively, and is, therefore, more representative regarding the provided information (Tversky and Kahneman, 1983). The first sequence, though, again is more probable than the second one since the latter includes the first one.

To what extent can natural frequencies help in both problems? Note that neither in the “Linda problem” nor in “rolling the dice” concrete probabilities are asked for.^2^ However, at least a “frequentist formulation” of both problems is possible, for instance: “Which option occurs most often?” In the Linda task, such a formulation does not seem possible at first sight, since the task is about a single event probability (Linda is only one person). Even in this case, though, one can imagine, for example, 200 people, who fit Linda’s description (Fiedler, 1988). Picturing these 200 people while asking oneself, how many are (1) bank tellers or (2) bank tellers and simultaneously active in the feminist movement, makes it easier to understand the task regardless of whether such 200 people exist or not (Fiedler, 1988).

Wedell and Moro (2007) investigated the effect of such frequentist questions in multiple similar scenarios (including rolling the dice), but found no systematic differences between probability and frequentist questions. Interestingly, already Inhelder and Piaget (1964) implemented a frequentist question for investigating their so-called *class-inclusion* *problem*. They concluded that children who are asked whether there are more red roses or roses in a bouquet often choose the answer “red roses,” although the latter ones clearly are included in the answer “roses.”

Note that in all examples so far only a *qualitative* comparison of P(A∩B) and P(A) was asked for. While Fiedler (1988) found increased performances based on a frequency question, Wedell and Moro (2007) did not. Also, Inhelder and Piaget (1964) did not identify a frequentist formulation as beneficial, which overall results in mixed evidence.

#### 2.3.2 Calculating P(A∩B) based on concrete given probabilities

**Figure 2:**
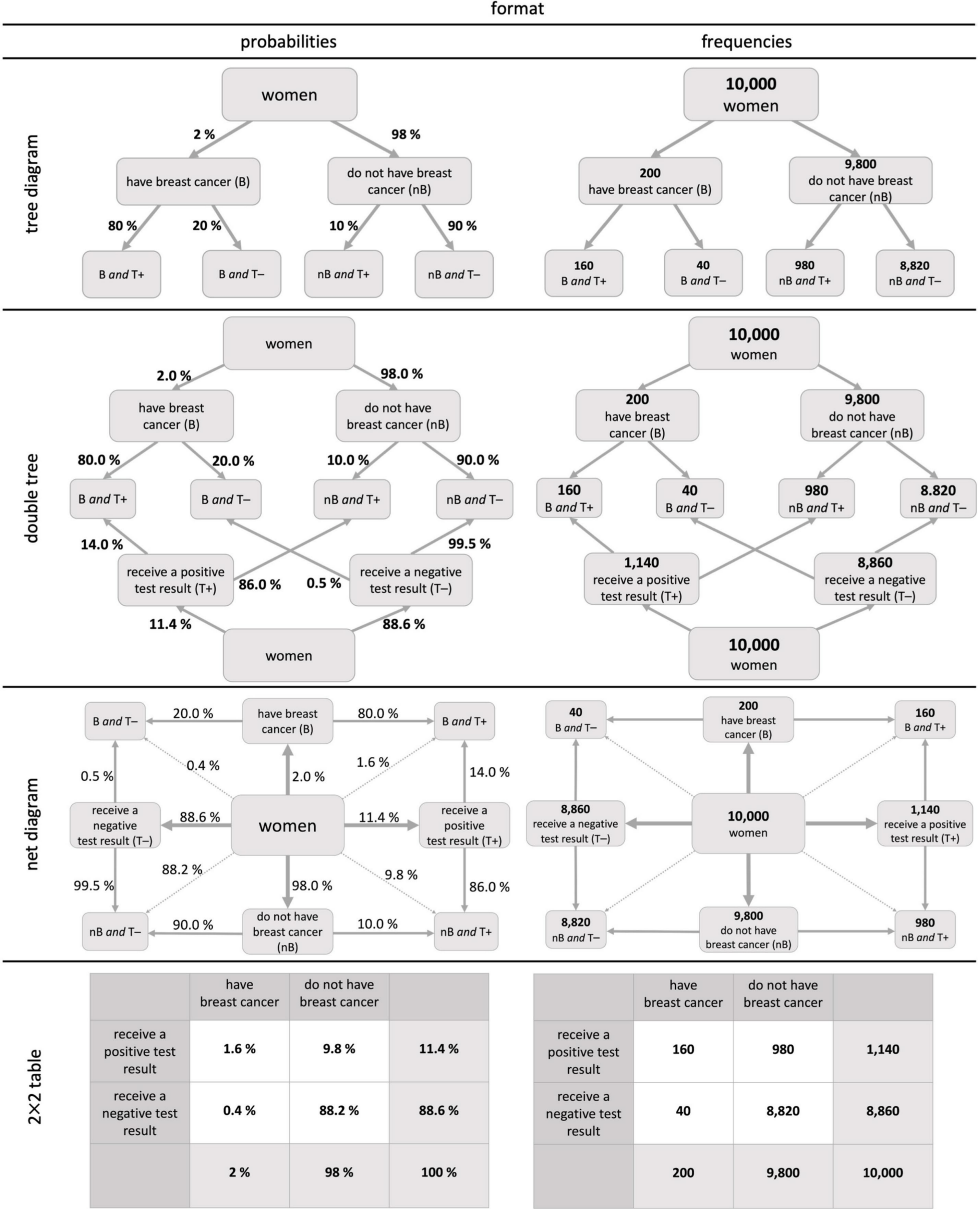
Visualizations of two binary events in the context of the mammography problem: Probability versions (left) and frequency versions (right).^3^

Basically, there are two options for displaying *concrete* probabilities that allow assessing a joint probability. One of them is presenting several concrete pieces of information in a *text* and the other one is to provide statistical information in *visualizations* (also see Figure 2).

Concerning a textual representation, the question arises, which pieces of information should or must be given to determine a correct joint probability answer. In the Bayesian reasoning paradigm both the given pieces of information *and* the specific question are predefined. Interestingly, based on the typical three given pieces of information in a “Bayesian text,” namely P(B), P(A|B), and $P(A|\bar{B}),$ not only the positive predictive value, but also all four joint probabilities can be calculated in principle. This set of information is, in so far, “complete” because it allows for the calculation of all 16 elementary probabilities.

It is important to note that for calculating *one specific* joint probability, i.e., P(A∩B), only two probabilities are needed (e.g., P(A) and P(B|A) or P(B) and P(A|B), respectively). Yet, if *all four* joint probabilities were asked for, more information would be necessary (for a case-by-case analysis see Stegmüller, 2020). For this reason, it is evident that providing a “Bayesian text” allows some generalization potential regarding the judgment of joint probabilities.

When asking for all four joint probabilities based on the mentioned set of given information P(B), P(A|B),$P(A|\bar{B}),$ the four types (P(A∩B),$P(\bar{A}\cap B),P(\bar{A}\cap \bar{B}),P(A\cap \bar{B}))$ see Table 1) require a different number of mental steps. Looking at Table 1, it becomes clear that, for the first type, all needed factors for answering this “joint question” are directly given in the “Bayesian text” (Figure 1), whereas, for the third type, even two counter probabilities have to be assessed first. In the frequency version, the first and the last type can be inferred by “skipping one level” and reading off the correct numbers only (for the example in Figure 1, e.g., 160 out of 10,000 and 980 out of 10,000, respectively), while the counter events need to be assessed first for the other two joint frequencies (e.g., 40 out of 10,000 and 8,820 out of 10,000, respectively).

**Table 1:** Information given or not given in the “Bayesian text” in both formats; each “X” requires an additional mental step.

	Joint question	Probability format			Frequency format
		Needed calculation	First factor	Second factor	Both needed absolute frequencies
Type 1	P(A∩B)	P(B) ⋅ P(A|B)	✓	✓	✓
Type 2	$P(\bar{A}\cap B)$	P(B) ⋅$P(\bar{A}|B)$	✓	X	X
Type 3	$P(\bar{A}\cap \bar{B})$	$P(\bar{B})$⋅$P(\bar{A}|\bar{B})$	X	X	X
Type 4	$P(A\cap \bar{B})$	$P(\bar{B})$⋅$P(A|\bar{B})$	X	✓	✓

Note that also the questions with the switched event order (e.g., P(B∩A)) have the same calculation steps as the listed ones (e.g., P(A∩B)).

✓: directly given; X: not directly given; probability/frequency of counter event needs to be inferred first.

A first attempt to ask for a joint probability based on such a “Bayesian text” was made by Binder et al. (2020), however, in this study, only one joint probability was asked for (type 2 in Table 1). Although there was no substantial frequency effect (see Table 2: “Bayesian text”), this finding cannot simply be transferred to the other three joint probabilities, since the different questions require a different number of mental steps (Table 1; Ayal and Beyth-Marom, 2014) and are, thus, not directly comparable.

**Table 2:** Results in previous studies with questions on joint probabilities.

Information format	Bruckmaier et al. (2019)		Binder et al. (2020)			
	Tree diagram	2 × 2 table	“Bayesian text”	Double tree	Net diagram	2 × 2 table
Probabilities	46%	96%	16%	16%	59%	78%
Natural frequencies	50%	79%	22%	48%	45%	52%

Another way to provide concrete probabilities that allow to assess a joint probability is to present them in a visualization. Figure 2 displays four visualizations that were already used for joint probability judgements in prior studies (yet, not systematically) based on the context and numbers given in Figure 1 (Bruckmaier et al., 2019; Binder et al., 2020). Note that the first two (tree diagram and double tree) display *conditional* but no *joint* probabilities. The opposite is true for the 2 × 2 table. Only the net diagram has the advantage of displaying *both* conditional and joint probabilities.

The tree diagram, the double tree, and the net diagram have a node-branch structure in which probabilities can be entered at the branches (Figure 2, left) and frequencies in the nodes (Figure 2, right). Nevertheless, frequencies and probabilities can, in principle, also be included simultaneously (imagine putting the left and the right visualization on top of each other), which makes it possible to depict both formats into the visualization at once (Binder et al., 2023). Thereby, the net diagram is the only visualization that can display all 16 probabilities. This versatility of the net diagram (i.e., all 16 probabilities and all 9 frequencies can be inserted), however, raised the concern that it would lead to a cognitive overload for students or study participants (Henze and Vehling, 2021). In 2 × 2 tables, cells normally *either* include probabilities *or* frequencies.

In both probability trees (simple and double), the answer to all joint questions cannot be read off directly but must be calculated first (e.g., in Figure 2: P(B∩T+) = P(B) ⋅ P(T +|B) = 2% ⋅ 80% = 1.6%). In the net diagram and the 2 × 2 table in probability format, only the correct numbers have to be read off, which results in fewer mental steps than in the tree diagrams. In the frequency format, however, all visualizations directly deliver the same information, since in each visualization, only the correct two numbers have to be combined without a calculation.

Table 2 presents previous results, when a joint probability question was asked explicitly based on these visualizations (Bruckmaier et al., 2019; Binder et al., 2020). While there seems to be no format effect in the “normal” tree diagram (Bruckmaier et al., 2019), natural frequencies appear to have a positive effect when placed in a double tree (Binder et al., 2020). Interestingly, in 2 × 2 tables, natural frequencies even seem to deteriorate the performance (see both studies in Table 2).

However, Bruckmaier et al. (2019) conducted an eye-tracking study with only 24 participants and Binder et al. (2020) focused predominantly on conditional probabilities (i.e., Bayesian reasoning).

In both studies, previously posed conditional probability questions might have framed participants toward thinking of conditional probabilities and, thereby, might have had an influence on the answer to the following joint probability question. Furthermore, in both studies, only one of the four possible joint probabilities was asked for, namely the one without the need to infer counter events first. Taken together, the findings in Table 2 must be interpreted very carefully.

In the present article, the understanding and assessing of joint probabilities and frequencies in situations with two binary events is examined for the first time systematically. Note that we are not primarily interested in which visualization is better than the other to foster understanding of joint probabilities. Rather, different visualization types have the potential to display statistical information in various ways and, thus, allow exploring possible format effects on a more differentiated level. In principle, we are, therefore, interested in potential interactions of a possible frequency effect with (a) the underlying representation of statistical information and (b) the type of probability question asked P(A∩B),$P(\bar{A}\cap B),P(\bar{B}\cap \bar{A}),P(\bar{B}\cap A).$ Both perspectives aim at generalizing possible frequency effects regarding the assessment of “joint information.”

## 3 Present approach

In the present study, we investigate people’s ability to assess concrete joint probabilities or frequencies based on various ways to represent statistical information. To study format effects, we considered five different “visualization types,” namely the “Bayesian text” (no visualization) and the four completely filled visualizations from Figure 2. Next to each visualization, no additional text with statistical information was given. Since each of the five visualization types (“Bayesian text,” 2 × 2 table, tree diagram, double tree, net diagram) can be equipped with both information formats (probability or natural frequency), we implemented 10 different stimuli. Based on all visualization types, we, furthermore, ask for all four possible joint probabilities or frequencies.

Our research question is:

RQ: What is the effect of information format (i.e., probabilities vs. natural frequencies) for assessing all four concrete joint probabilities/frequencies when statistical information is presented as

a. “Bayesian text,” i.e., the three pieces of information (base rate, sensitivity, and false-alarm-rate) typically presented in Bayesian reasoning tasks are provided in textual form

or in a completely filled visualization (Figure 2), namely as

b. tree diagram
c. double tree
d. net diagram
e. 2 × 2 table?

Furthermore, we want to know whether the type of joint probability $\left(P(A\cap B),P(\bar{A}\cap B),P(\bar{B}\cap \bar{A}),P(\bar{B}\cap A)\right)$ that was asked substantially changed participants’ performance.

### 3.1 Hypotheses regarding research question (a) Bayesian text

In the probability version, answers need to be calculated, for example, by applying the multiplication rule (e.g., “2% ⋅ 80%”). In the natural frequency format, most absolute frequencies that must be combined for the correct answer are already available (depending on the type of question; see Table 1). For instance, in the “Bayesian text” in Figure 1, the first two provided natural frequencies (“200 out of 10,000” and “160 out of 200”) have to be combined correctly to receive the answer “160 out of 10,000.” Note that in both formats some of the given information has to be ignored. Since a calculation with probabilities seems to be more difficult than choosing and combining the right frequencies, we assume—in contrast to the results of Binder et al. (2020)—a substantial format effect here. Consequently, a natural frequency formulation should enhance the performance for questions on joint probabilities. Moreover and regarding the four types (Table 1), it is expected that the more counter events from the “Bayesian text” have to be inferred first, the less correct solutions will be given.

### 3.2 Hypotheses regarding research question (b) - (e) visualizations

Neither in the tree diagram nor in the double tree, joint probabilities are displayed, meaning that they must be calculated (e.g., by the multiplication rule). In the frequency versions of both tree diagrams, the two relevant absolute frequencies can be read off directly and only have to be combined, which is why we expect a positive format effect here. All four joint probabilities can be directly read off from the net diagram and the 2 × 2 table, so high solution rates can be expected even in probability versions (these performances might be probably higher in the 2 × 2 table because less other possibly interfering probabilities are displayed as compared to the net diagram). According to Bruckmaier et al. (2019) and Binder et al. (2020), even a reverse format effect might be expected for the net diagram and the 2 × 2 table, since *two* relevant frequencies have to be identified first and then combined correctly.

In sum, concerning (b) and (c), we expect a format effect in favor of natural frequencies, while concerning (d) and (e), we expect no or even an opposite format effect.

Since in each implemented visualization, all statistical information is presented in a “symmetrical way” and no counter events have to be inferred, no differences are expected regarding the different type of probability question. Yet, the various types of joint probabilities still differ in a linguistic way since the number of negations in the question varies.

## 4 Method

### 4.1 Design

Participants had to work on two different contexts (i.e., mammography problem and economics problem; the first adapted from Eddy, 1982, and the second from Ajzen, 1977). In each context, they had to assess all four possible joint probabilities or frequencies. So, every participant had to work on eight tasks.

The study design (see Table 3) includes three factors (information format, visualization type, and context). This leads to a 2 × 5 × 2 design:

- Factor 1: information format: probabilities vs. natural frequencies
- Factor 2: visualization type: “Bayesian text” (no visualization) vs. 2 × 2 table vs. tree diagram vs. double tree vs. net diagram
- Factor 3 (not a factor of interest): context: mammography vs. economics problem

**Table 3:** Study design.

First context processed*			Second context processed*		
Probabilities	×	“Bayesian text”	Probabilities	×	“Bayesian text”
		Tree diagram			Tree diagram
		Double tree			Double tree
Natural frequencies		Net diagram	Natural frequencies		Net diagram
		2 × 2 table			2 × 2 table
All four possible joint questions (order of the events within a question was varied)			All four possible joint questions (order of the events within a question was varied)		

Each participant worked on both contexts. If the first context was presented, e.g., in natural frequencies and a net diagram, these both conditions were excluded for the second context.

*= order of contexts, formats, and the two visualization conditions were counterbalanced.

Factor 1 is the main factor of interest by considering possible interactions with factor 2, while factor 3 was not a factor of interest but only implemented for mutual validation. Furthermore, each participant answered all four possible joint questions $\left(P(A\cap B),P(\bar{A}\cap B),P(\bar{B}\cap \bar{A}),P(\bar{B}\cap A)\right)$ in both contexts. To control for effects of the event order (i.e., asking for P(A∩B) vs. asking for P(B∩A)), two questions always first included the event A (e.g., getting a positive test result or not) and the other two the event B (e.g., having breast cancer or not).

### 4.2 Instruments and administration

For each context, 10 stimuli were constructed according to Table 3. In the testlets, one context (for both contexts see Table 4) per participant was always presented in natural frequencies and the other one in probabilities. If the first context processed was based on one out of five visualization types (“Bayesian text,” tree diagram, double tree, net diagram, 2 × 2 table), the second context was presented in one out of the remaining four visualization types. Thus, the instruments were systematically constructed from the modules in Table 3. The rule was: If a participant worked on context X, information format Y, and visualization type Z, exactly these three conditions were forbidden for the second context processed. Every context comprised all four possible joint questions.

**Table 4:** Stimuli that emerged by systematically varying factors 1–3 (see Figure 2 for the visualizations).

		Mammography problem		Economics problem	
		Probabilities	Natural frequencies	Probabilities	Natural frequencies
Cover story		Imagine you are a reporter for a women’s magazine and you want to write an article about breast cancer. As a part of your research, you focus on mammography as an indicator of breast cancer. You are especially interested in the question of what it means if a woman has a positive result (which indicates breast cancer) in such a medical test. Please answer the following questions using the statistical information provided below:		Imagine that you are interested in the question of whether students at a boys’ school are more likely to choose economics courses or other courses at their school. For this purpose, you refer to a study conducted by the school psychology service on the connection between personality traits in students and the choice of subjects. Please answer the following questions using the statistical information provided below:	
Statistical information (visualization type)	“Bayesian text”	The probability that a woman who goes for a routine screening has breast cancer is 2%. If a woman who goes for a routine screening has breast cancer, the probability that she will get a positive test result is 80%. If a woman who goes for a routine screening does not have breast cancer, then the probability that she will still get a positive test result is 10%.	200 out of 10,000 women who go for a routine screening have breast cancer. Out of 200 women who go for a routine screening and have breast cancer, 160 get a positive test result. Out of 9,800 women who go for a routine screening and do not have breast cancer, 980 still get a positive test result.	The probability that a student attends the economics course is 32%. If a student attends the economics course, the probability that he is career-oriented is 64%. If a student does not attend the economics course, the probability that he is still career-oriented is 60%.	320 out of 1,000 students attend the economics course. Out of 320 students who attend the economics course, 205 are career-oriented. Out of 680 students who do not attend the economics course, 408 are still career-oriented.
	Visualization	2 × 2 table with probabilities, or	2 × 2 table with natural frequencies, or	2 × 2 table with probabilities, or	2 × 2 table with natural frequencies, or
		Tree diagram with probabilities, or	Tree diagram with natural frequencies, or	Tree diagram with probabilities, or	Tree diagram with natural frequencies, or
		Double tree with probabilities, or	Double tree with natural frequencies, or	Double tree with probabilities, or	Double tree with natural frequencies, or
		Net diagram with probabilities	Net diagram with natural frequencies	Net diagram with probabilities	Net diagram with natural frequencies
1^st^ question P(A∩B)		What is the probability that a woman who goes for a routine screening will get a positive test result *and* has breast cancer?	How many of the women who go for a routine screening get a positive test result *and* have breast cancer?	What is the probability that a student is career oriented *and* chooses the economics course?	How many of the students are career oriented *and* choose the economics course?
2^nd^ question $P(\bar{A}\cap B)$		What is the probability that a woman who goes for a routine screening will get a negative test result *and* has breast cancer?	How many of the women who go for a routine screening get a negative test result *and* have breast cancer?	What is the probability that a student is not career oriented *and* chooses the economics course?	How many of the students are not career oriented *and* choose the economics course?
3^rd^ question $P(\bar{B}\cap \bar{A})$		What is the probability that a woman who goes for a routine screening does not have breast cancer *and* will get a negative test result?	How many of the women who go for a routine screening do not have breast cancer *and* get a negative test result?	What is the probability that a student does not choose the economics course *and* is not career oriented?	How many of the students do not choose the economics course *and* are not career oriented?
4^th^ question $P(\bar{B}\cap A)$		What is the probability that a woman who goes for a routine screening does not have breast cancer *and* will get a positive test result?	How many of the women who go for a routine screening do not have breast cancer *and* get a positive test result?	What is the probability that a student does not choose the economics course *and* is career oriented?	How many of the students do not choose the economics course *and* are career oriented?
Answer format		_________ (please specify to one decimal place)	____out of ______	_________ (please specify to one decimal place)	____out of ______

Besides the eight joint probability or frequency judgements, several covariates were collected from all participants (see 4.3): level of education (“Fachsemester”), grade point average from high school (German “Abiturnote”), the highest school degree, the field of study, gender, and age.

We varied the first three factors between participants (yielding 160 different testlets) and gave two participants that were sitting next to each other always different contexts for the first task. The two different scenarios (Table 4) were handed out one after the other to track the order of processing. The participants did not have a time limit, but they could use as much time as they wanted to. It took them between 5 and 25 minutes to complete all eight tasks. Further, they were given calculators since the study was on their understanding of the tasks and not on their ability to calculate.

### 4.3 Participants

Data analysis was based on *N* = 335 students who were examined during university classes in Bavaria (Germany) in the year 2022. Students of social work (*N* = 251), biomedical engineering (*N* = 53), and business classes (*N* = 31) participated. *N* = 271 students were female, *N* = 62 male, and *N* = 2 nonbinary. The average age was *M* = 22.5 (*SD* = 4.0).

The study was carried out in accordance with the Research Ethics Standards of the university. Students were informed that their participation was voluntary, and anonymity was guaranteed. Initially, we had *N* = 339 students attending, but only *N* = 335 were considered for the analysis because two withdrew their consent and two more mentioned that they did not really think about the tasks and did not put any effort in trying to solve them.

Note that in German schools, only 2 × 2 tables (either filled with probabilities or frequencies) and tree diagrams (only with probabilities) were taught, so students probably were familiar with these types of visualizations.

### 4.4 Coding

An overview of the correct answers for each of the eight questions (for both contexts and both formats) is given in Table 5. For the probability versions, the correctness of a response is classified according to whether the participant gave the correct answer within a certain interval of rounding (± 0.1%). For the natural frequency version, both absolute frequencies had to be correct (no rounding occurs). Interrater reliability between two raters was calculated based on 15% of the data and yielded a Cohens Kappa of κ = 1 (Cohen, 1960), therefore answers could be coded with a maximum of objectivity.

**Table 5:** Coding of the correct answers regarding all questions.

		Probabilities		Natural frequencies
		Correct answer	Interval in which answers were coded correct	Both absolute numbers must be exact
Mammography	Having breast cancer joint with a positive test result	1.6%	[1.5%; 1.7%] or a decimal fraction in [0.00; 0.02]	The correct answer is 160 out of 10,000.
	Having breast cancer joint with a negative test result	0.4%	[0.3%; 0.49%] or a decimal fraction in [0.00; 0.0049]	The correct answer is 40 out of 10,000.
	Not having breast cancer joint with a negative test result	88.2%	[88.1%; 88.3%] or a decimal fraction in [0.88; 0.89]	The correct answer is 8,820 out of 10,000.
	Not having breast cancer joint with a positive test result	9.8%	[9.7%; 9.9%] or a decimal fraction in [0.09; 0.10]	The correct answer is 980 out of 10,000.
Economics problem	Choosing the economics course joint with interest in a career	20.5%	[20.4%; 20.6%] or a decimal fraction in [0.20; 0.21]	The correct answer is 205 out of 1,000.
	Choosing the economics course joint with no interest in a career	11.5%	[11.4%; 11.6%] or a decimal fraction in [0.10; 0.12]	The correct answer is 115 out of 1,000.
	Not choosing the economics course joint with no interest in a career	27.2%	[27.1%; 27.3%] or a decimal fraction in [0.27; 0.30]	The correct answer is 272 out of 1,000.
	Not choosing the economics course joint with interest in a career	40.8%	[40.7%; 40.9%] or a decimal fraction in [0.40; 0.41]	The correct answer is 408 out of 1,000.

The problem of different rounding only occurs in the probability version, which is why only in these versions (and not in the frequency versions) answers within a certain interval were accepted. If we allowed the same interval for natural frequencies, though, nothing in the coding would change.

## 5 Results

### 5.1 Descriptive results regarding the four types of questions

Unexpectedly, there were almost no substantial differences regarding the special type of joint probability that was asked $(P(A\cap B),P(\bar{A}\cap B),P(\bar{B}\cap \bar{A}),P(\bar{B}\cap A);$ always in this order). In Supplement S1, all descriptive results are displayed for each single stimulus. Across all versions, the type of question asked and, thus, the number of counter events that first had to be assessed as well as the number of negations in the question do not seem to make a substantial difference.

**Figure 3:**
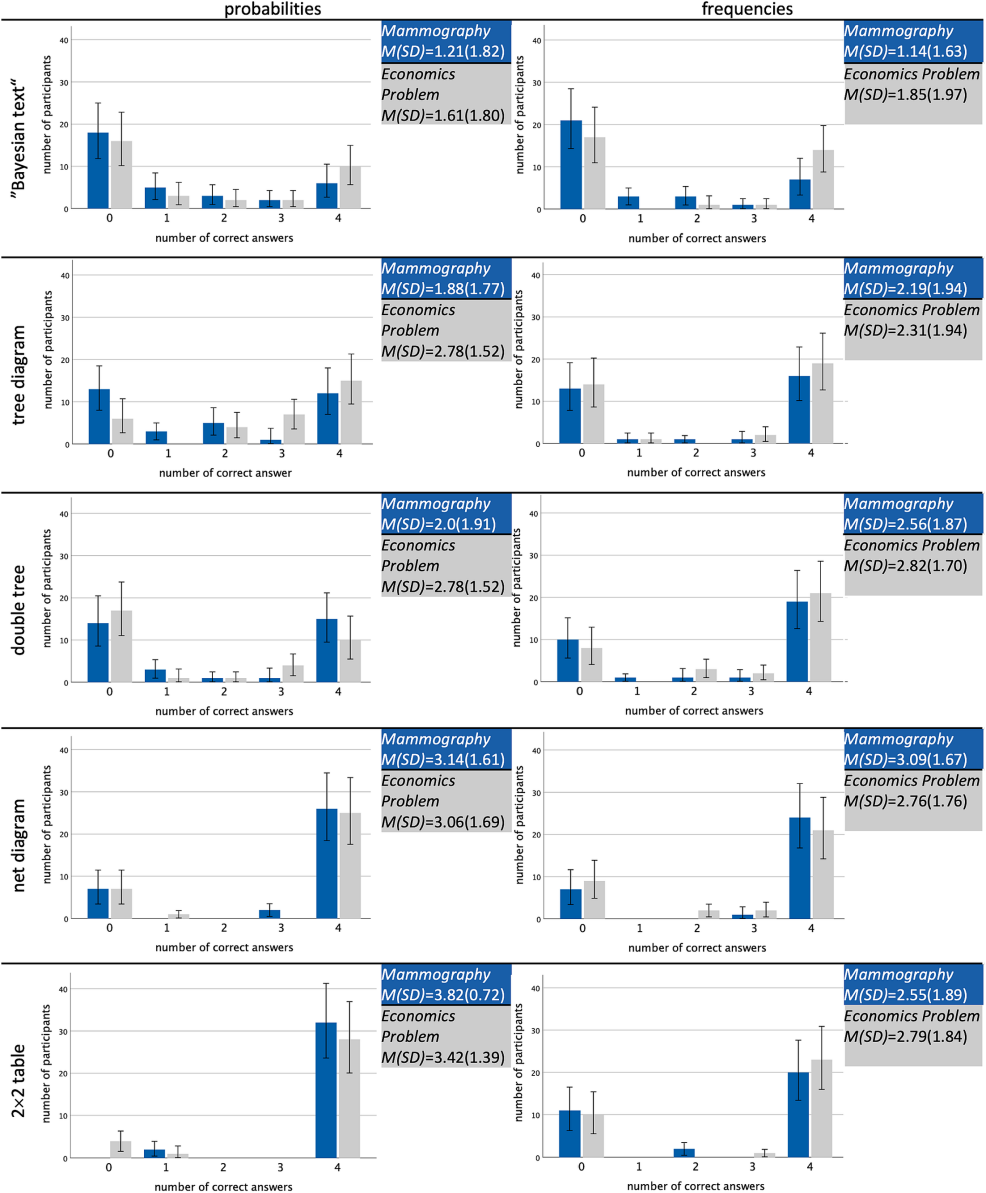
Overview of the absolute numbers of participants achieving no, one, two, three, or all four correct answers regarding all four types (Table 1), separated for all 20 stimuli.

Another perspective on this fact is given by Figure 3, which illustrates the number of correct joint inferences (0–4). According to the bar diagrams, participants rather predominantly answered *none* or *all* of the four questions correctly. Thus, they either understood how to calculate or read off the answer or they did not at all, regardless of which information format was given. In the following, we will, therefore, report results aggregated across the four joint questions.

### 5.2 Results regarding research questions (a)–(e)

**Figure 4:**
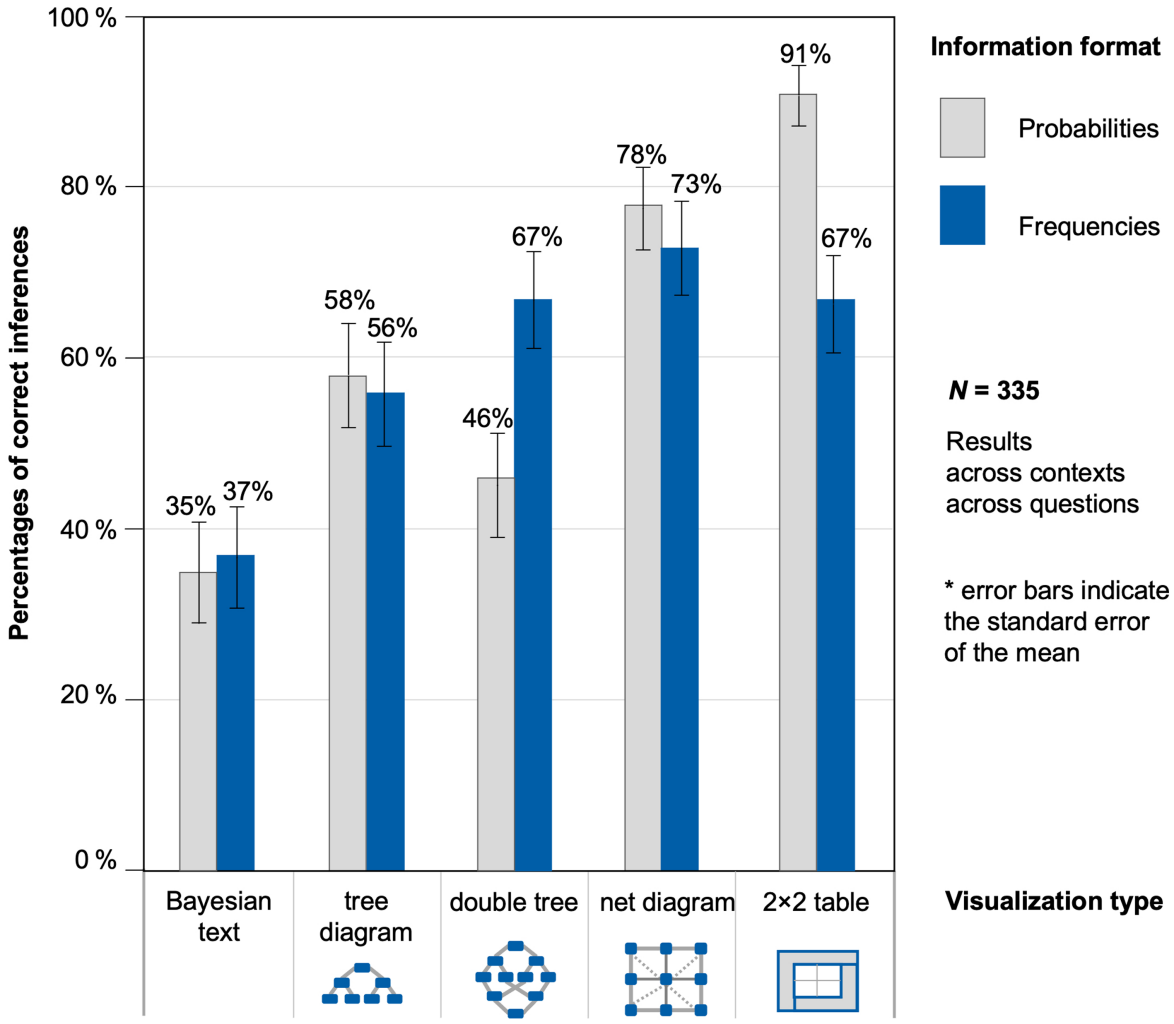
Percentages of correct inferences, separated by information format and visualization type across both contexts and across all four joint probability questions.

There seems to be a highly differential format effect regarding each visualization type (Figure 4). Because the response patterns in both contexts were very similar, Figure 4 displays the results across contexts. By considering the visualizations separate from each other, two opposite results can be observed already at a descriptive level: the expected frequency effect for the double tree and a reverse effect for the 2 × 2 table in which the probabilities lead to better performances.

To analyze the effects of information format, visualization type, and their interaction effects by means of inferential statistics, we estimated a generalized linear mixed model (GLMM) with a logit link function to predict the probability that participants solve a question for joint probabilities or frequencies correctly (as a binary dependent variable with 0 = wrong, 1 = correct). We decided for a *mixed* analysis and against a, for instance, generalized linear model (i.e., a logistic regression) due to our between-within-subject design since each participant solved several tasks. To take this aspect into account, we modeled a generalized linear *mixed* model with the participants’ ID as a random factor, so that the participant-specific error is also modeled (Figure 3 shows dependencies between the responses). In the generalized linear mixed model, we specified the probability version of the “Bayesian text” as the reference category and included the possible explanatory factors “frequencies,” on the one side and, on the other side, “tree diagram,” “double tree,” “net diagram,” and “2 × 2 table” via dummy coding. Furthermore, since the performance in the different formats was expected to vary depending on the visualization type, four interaction terms *visualization × format* were modeled as fixed effects.

Because the answers of the participants were dependent on each other (Figure 3) and to exclude sequence effects, we also controlled for the fact that one participant worked on more than one task. Specifically, we implemented participants’ ID (w_1_) and the order of the questions: 1^st^, 2^nd^, 3^rd^, 4^th^, 5^th^, 6^th^, 7^th^, and 8^th^ (w_2_) as random factors in the generalized linear mixed model:

$\begin{array}{l} \hat{y}=\beta_{0}+\beta_{1}\cdot \text{frequencies}+\beta_{2}\cdot \text{tree diagram}\\ \;+\beta_{3}\cdot \text{double tree}+\beta_{4}\cdot \text{net}\;\text{diagram}+\beta_{5}\cdot 2\times 2\;\text{table}\\ \;+\beta_{6}\cdot \text{tree diagram}\times \text{frequencies}+\beta_{7}\cdot \text{double tree}\times \text{frequencies}\\ \;+\beta_{8}\cdot \text{net}\;\text{diagram}\times \text{frequencies}+\beta_{9}\cdot 2\times 2\;\text{table}\times \text{frequencies}\\ \;+w_{1}+w_{2} \end{array}$

The regression coefficient for the frequencies was significantly negative (Table 6), which means that, in the “Bayesian text,” tasks in probabilities are better solved than the ones in natural frequencies. This “probability effect” also holds true for the 2 × 2 table and the net diagram but does not become substantially bigger as can be seen from the regarding interactions that are not significant. In contrast, for the tree diagram and the double tree, this interaction was significantly positive, meaning that the negative format effect observed in the “Bayesian text” is outweighed in these two versions. As a side effect of the findings, we can observe that each visualization compared to the text version—except the double tree—has significant regression coefficients, which means that all of these visualizations in the probability version improved participants’ performance. All fixed effects of the model explain 16.3% of the variance, whereas fixed and random effects together explain 75.9% of the variance.

**Table 6:** Regression coefficients for information format, visualization type, and their interactions.

		Estimate	*SE*	*z*	*p*
β_0_	Intercept	−0.27	0.30	−0.90	0.37
**β**_**1**_	**Frequencies**	**−1.18**	**0.40**	**−2.97**	**0.003**
**β**_**2**_	**Tree diagram**	**0.71**	**0.34**	**2.08**	**0.04**
β_3_	Double tree	−0.05	0.37	−0.14	0.89
**β**_**4**_	**Net diagram**	**3.46**	**0.38**	**9.00**	**< 0.001**
**β**_**5**_	**2 × 2 table**	**3.75**	**0.45**	**8.30**	**< 0.001**
**β**_**6**_	**Tree diagram × frequencies**	**2.26**	**0.58**	**3.89**	**< 0.001**
**β**_**7**_	**Double tree × frequencies**	**2.79**	**0.61**	**4.54**	**< 0.001**
β_8_	Net diagram × frequencies	−0.86	0.58	−1.50	0.13
β_9_	2 × 2 table × frequencies	−0.17	0.63	−0.27	0.79

Note that bold regression coefficients are significant at *α* = 0.05.*R*^2^_marginal_ = 16.3%, *R*^2^_conditional_ = 75.9%.

If the question type $\left(P(A\cap B),P(\bar{A}\cap B),P(\bar{B}\cap \bar{A}),P(\bar{B}\cap A)\right)$ is additionally implemented in the model (not displayed in Table 6), it can be observed that none of the other question types is solved correctly significantly rarer than the (easiest) question for P(A∩B). Moreover, the implementation of this variable, as well as other covariates such as age, gender, level of education, mathematics grade, and school degree, does not lead to substantial changes in the results presented.

Note that some of the results displayed in Table 6 at first seem to contradict the results in Figure 4. Concerning the “Bayesian text,” for example, there was a descriptive advantage of frequencies in Figure 4, while, with inferential statistics, the outcome is the opposite. The results differ because we controlled for order and ID in the GLMM, which we did not in the descriptive results. Of course, we varied all versions systematically when collecting the data, but, obviously, there are still “group” effects. This demonstrates the need for multi-level modeling since these more precise results cannot be obtained from the descriptive results alone.

## 6 Discussion

### 6.1 Summary

In the present study, we systematically investigated participants’ assessment of concrete joint probabilities in Bayesian reasoning situations. In the theoretical part, we distinguished between paradigms that ask for a qualitative comparison of P(A) and P(A∩B) and paradigms in which, principally, the whole “Bayesian situation” consisting of 16 probabilities is considered and, therefore, (all) joint probabilities can be assessed. After summarizing pertinent literature, we concluded that the evidence on a possible format effect with respect to joint probabilities is mixed.

In the empirical part of the paper, we reported a study with a 2 × 5 × 2 design with the factors information format (probabilities vs. natural frequencies), visualization type (“Bayesian text” vs. tree diagram vs. double tree diagram vs. net diagram vs. 2 × 2 table), and context (mammography vs. economics problem). Furthermore, each participant answered all four joint questions $(P(A\cap B),P(\bar{A}\cap B),P(\bar{B}\cap \bar{A}),P(\bar{B}\cap A)).$ Information format was the main factor of interest, and it was investigated which representation of a Bayesian situation shows which format effect.

First of all, looking at interactions between visualizations and information format, there were some opposite format effects. While tasks with probabilities improved participants’ performance in three visualization conditions (“Bayesian text,” net diagram, and 2 × 2 table), this effect cannot be observed with tree diagrams and double trees. Second and compared to the “Bayesian text”, participants’ performance improved with the probability versions of the tree diagram, the net diagram, and the 2 × 2 table. However, it was not of our interest *per se* to examine which visualization improves the performance the most. Nevertheless, we found tendencies that suggest which visualizations should be used when explaining situations with joint probabilities, which will be shown in the following section.

### 6.2 Open questions: Linda and Sally Clark

Although we did not explicitly contribute to these two situations by our experimental setting, let us, nevertheless, recapitulate these situations shortly. With respect to the visualizations in Figure 2, Linda as well as Sally Clark “happen” in only one branch (or in one column of a 2 × 2 table) because only P(A) and P(A∩B) are considered, which are depicted in one “line of branches.” The difference between both situations is that Sally Clark has a stronger sequential structure because the second child always succeeds the first one.

#### 6.2.1 Linda

**Figure 5:**
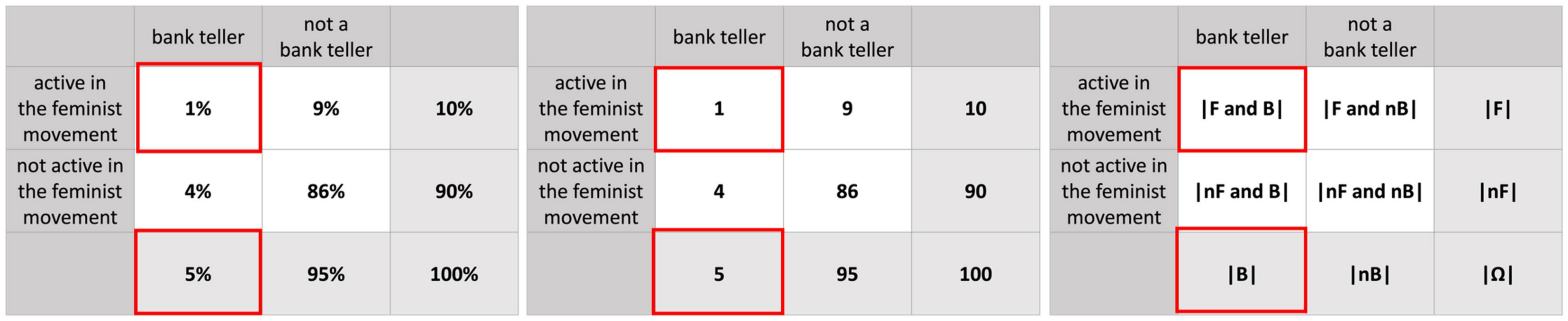
Visualization of the Linda version with 2 × 2 tables (probabilities, frequencies, abstract).

Our results would suggest explaining the Linda problem with a 2 × 2 table in probability format (left in Figure 5). So, it might become obvious that it is more probable to be a bank teller than to be a bank teller *and* to be active in the feminist movement, since 1% is smaller than 5% (which, of course, stays true for any other chosen imaginary numbers).

Comparing the 2 × 2 tables in probability and frequency format, in the latter one (center of Figure 5), whole persons and no percentages appear (see also, for example, Brase et al., 1998). This is why the 2 × 2 table with frequencies also seems to be rather intuitive. Indeed, to answer the Linda problem, in both tables, the same two cells have to be compared. Fiedler (1988) could foster his participants’ insight by letting them imagine 200 women fitting Linda’s description but without providing the other numbers. In any case, it must be noted that for answering the Linda question, marginal probabilities or frequencies (i.e., P(A)) have to be considered in addition, but the understanding of them was not subject of our study.

Perhaps the visualization of the general situation (right in Figure 5) in which no imaginary concrete numbers are given, would also enhance the performance in the Linda problem. The general 2 × 2 table would be more analog to the initial problem (no numbers are given) and it can be easily transferred into a filled-out version by, e.g., requesting the participants to complete the table with imaginary numbers. Thereby, it could either result in a probability or in a natural frequency version, so, alternatively, the abstract 2 × 2 table might be a good starting point for teaching in school.

#### 6.2.2 Sally Clark

**Figure 6:**
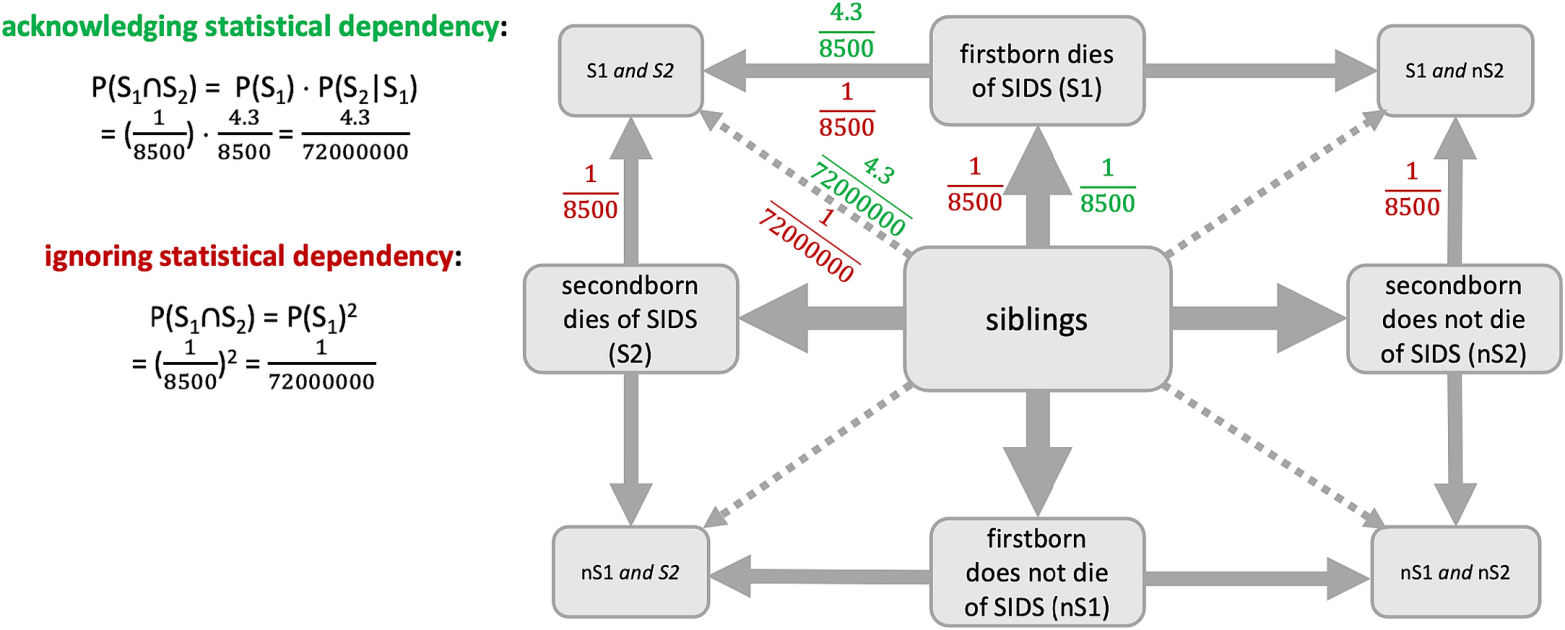
Visualization of the two situations (correct = acknowledging = green, false = ignoring = red) in the case of Sally Clark.

In the case of Sally Clark, information may be visualized in a tree diagram because of the sequential character of this situation. However, because our results would suggest an advantage of the net diagram and because this sequential character is served by the node-branch-structure, we display the net diagram here (Figure 6). In this visualization, joint probabilities can additionally be included. The red numbers show the situation that was wrongly assumed in court first, while the green numbers show the actual situation. The probability that the 2^nd^ child dies of SIDS (S_2_), if the 1^st^ child already died of SIDS (S_1_), is 4.3 times as likely as the probability that the 1^st^ child dies of SIDS (Glinge et al., 2023). In the case of Sally Clark, this would result in a probability of $\frac{1}{8500}$ ⋅ $\frac{4.3}{8500}$ = $\frac{4.3}{72000000}$ ≈ 0.000006%. Although the disregard of the stochastic dependency is often named as the reason for the misjudgment, the calculation shows that this cannot be the only reason since the probability is still very small. The mistrial, in fact, also ignored, for example, that even a very small probability never is equal to 0% and, thus, *does happen* sometimes (Colmez and Schneps, 2013). The medical expert, Roy Meadow, furthermore, assumed that mothers kill their children more often than one might think and, therefore, made this very clear as an expert during trial, which made people—and the jury—think that Clark killed her children (Colmez and Schneps, 2013). This shows that people thought to understand the situation, but apparently not all of them did.

### 6.3 Limitations and future research

Since we chose typical Bayesian contexts, we might have caused priming toward conditional probabilities among participants, although we did not ask for a conditional probability at any time. By taking the mammography context, for example, most people want to know what a positive or negative test result actually means and not how many people receive a positive test result *and* have breast cancer. Furthermore, in the text version, only the base rate, the sensitivity, and the false-positive-rate—the pieces of information that are typically given in Bayesian inference tasks—were given. This information might prime questions for conditional probabilities and not for joint probabilities. However, the economics context does not lead to a certain kind of question, which mitigates this claim. Still, we might have triggered different assumptions of the participants (e.g., the need for a conditional probability), which might have led to specific errors like answering with a conditional probability.

Furthermore, some participants might have also wondered why we “just” asked for *all four* joint probabilities and have not included conditional probability questions. Moreover, the fact that the visualizations included much more information might have made some participants evaluate their answers as “too easy,” which could have made them change their initial answer. By including only joint probabilities, we also cannot judge the format effect regarding marginal probabilities.

Future research could look more deeply into variations. At first, it would be interesting to vary the given pieces of information (especially in the textual version). Then, it would also be interesting to implement further contexts—especially ones that make perfectly sense concerning joint probabilities (e.g., gambling).

In addition, note that the efficacy of natural frequencies always also depends on more factors than the ones mentioned above: Ayal and Beyth-Marom (2014) showed that if the presented and requested format is not compatible (e.g., the information is in probabilities and the question in natural frequencies), the performance is lower than, for example, if both are in probabilities. However, highest performance levels can be observed, if information is presented in natural frequencies and participants *also work* with natural frequencies instead of translating them “back” into probabilities (Weber et al., 2018; Feufel et al., 2023). It also has an impact on the performance, whether the given information and the question are “aligned”, which means that the presented and requested information should be attached to the same subset (Tubau et al., 2019; Tubau, 2022; Brose et al., 2023). Furthermore, the performance also improves if the task format is formulated “explicitly” (the intersecting set is explicitly named, i.e., “How many of the positive tested women are ill and test positive?”) instead of “implicitly” (i.e., “How many of the positive tested women are ill?”; Böcherer-Linder et al., 2018). Future research should also consider these factors to be able to derive conclusions about their effect on joint probabilities.

Finally, we want to propose a fifth extension of Bayesian reasoning, namely, to explicitly address *all* possible 16 probabilities in future research. There are *eight* conditional probabilities; two of them are just complemented probabilities of the given sensitivity and false-alarm-rate. All four inverse conditional probabilities, nevertheless, belong to the full situation. From a mathematical viewpoint, all 16 probabilities are equally relevant and, furthermore, at school, of course, all of them are taught.

### 6.4 Conclusion

Our answer to the question “How general is the natural frequency effect?” is: There is no general statement possible concerning questions for joint probabilities. Whether natural frequencies improve participants’ performance in joint probability tasks highly depends on the way the statistical information is presented.

## Data availability statement

The data of the study can be found here: https://epub.uni-regensburg.de/54717/1/Datensatz_open.xlsx.

## Ethics statement

Ethical approval was not required for the studies involving humans in accordance with the local legislation and institutional requirements. The studies were conducted in accordance with the local legislation and institutional requirements. The participants provided their written informed consent to participate in this study.

## Author contributions

NS: Conceptualization, Data curation, Formal analysis, Funding, Investigation, Methodology, Project administration, Validation, Visualization, Writing – original draft, Writing – review & editing. KB: Conceptualization, Formal analysis, Methodology, Validation, Visualization, Writing – review & editing. SK: Conceptualization, Methodology, Validation, Writing – review & editing.

## Footnotes

^1^Of course, there are also the trivial probabilities P(∅) and P(Ω) as well as all probabilities regarding set unions, e.g., P(A∪B). An extensive overview and discussion of all possible cases can be found in Neth et al. (2021).

^2^Even though neither probabilities are given nor asked for explicitly, in “rolling the dice,” the probability of all three sequences can be calculated concretely: P(RGRRR) ≈ 0.82%, P(GRGRRR) ≈ 0.54% and P(GRRRRR) ≈ 0.27%.

^3^Note that because of the plural “women” in our probability trees (e.g., in Figures 1, 2) these trees are basically percentage trees. However, since research in Bayesian reasoning mostly distinguishes between probability and frequency format, we call them probability trees.
