# Corrigendum: How general is the natural frequency effect? The case of joint probabilities

**DOI:** 10.3389/fpsyg.2024.1515434

**Published:** 2024-11-19

**Authors:** Nathalie Stegmüller, Karin Binder, Stefan Krauss

**Affiliations:** ^1^Mathematics Education, Faculty of Mathematics, University of Regensburg, Regensburg, Germany; ^2^Mathematics Education, Institute of Mathematics, Ludwig Maximilian University Munich, Munich, Germany

**Keywords:** joint probabilities, Bayesian reasoning, natural frequencies, visualization, net diagram

In the published article, there was an error in Figure 2 as published. In the left net diagram, it said “B and T+” (right, bottom), although it should be “nB and T+”. The corrected Figure 2 and its caption appear below.

**Figure 2:**
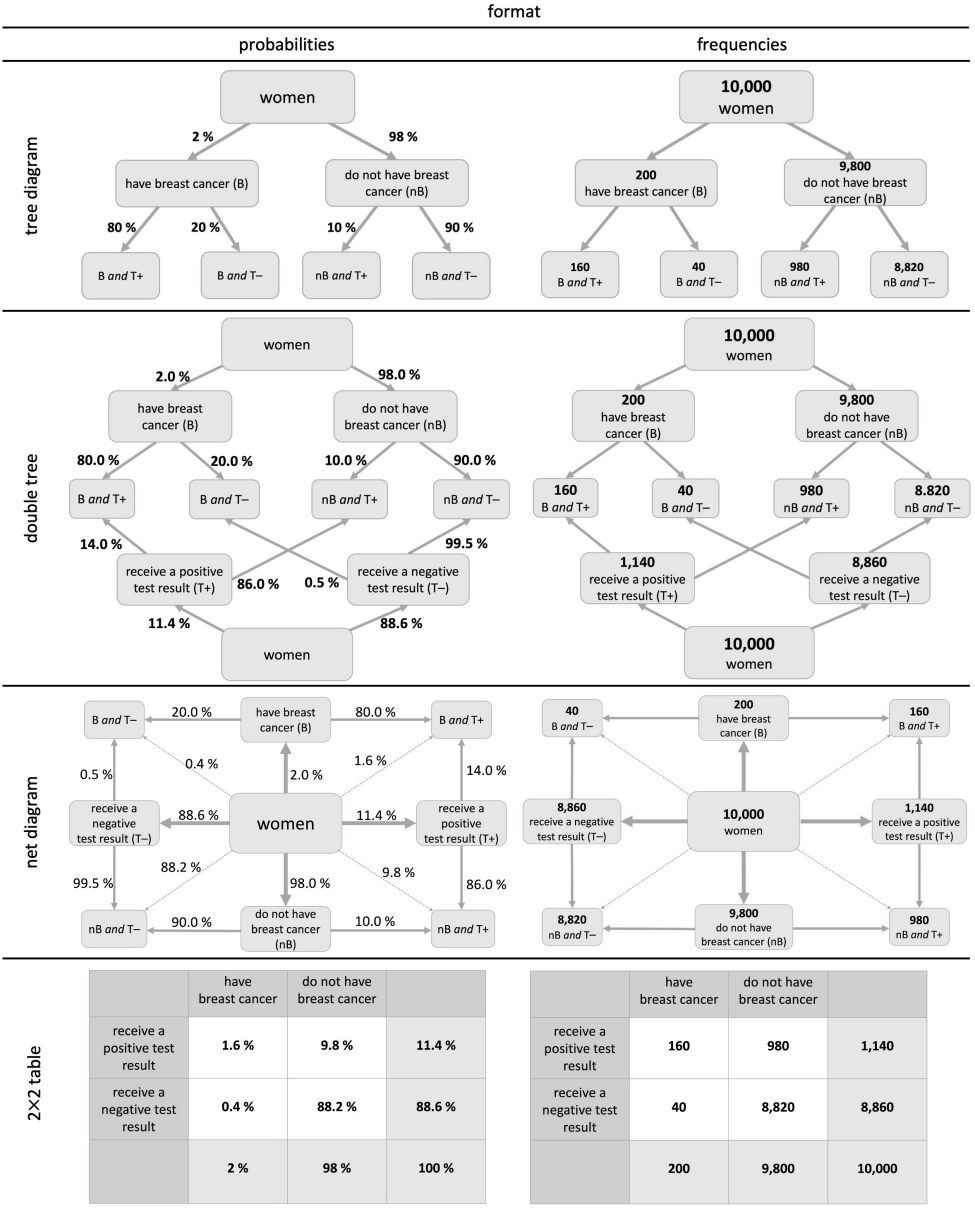
Visualizations of two binary events in the context of the mammography problem: Probability versions (left) and frequency versions (right).^3^

The authors apologize for this error and state that this does not change the scientific conclusions of the article in any way. The original article has been updated.

## Footnotes

^3^Note that because of the plural “women” in our probability trees (e.g., in Figures 1, 2) these trees are basically percentage trees. However, since research in Bayesian reasoning mostly distinguishes between probability and frequency format, we call them probability trees.

