# Supplementary material for: How general is the natural frequency effect? The case of joint probabilities
Source: Front Psychol. 2024 Apr 10;15:1296359. doi: 10.3389/fpsyg.2024.1296359 (PMC11040332; doi:10.3389/fpsyg.2024.1296359)
Supplement: Supplementary file 1 [file Table_1.pdf]

Supplement S1: Descriptive results for each single type of joint probability included in the 20 stimuli.

	probabilities								natural frequencies							
	mammography				economics problem				mammography				economics problem			
	$A \cap B$	$\bar{A} \cap B$	$\bar{B} \cap \bar{A}$	$\bar{B} \cap A$	$A \cap B$	$\bar{A} \cap B$	$\bar{B} \cap \bar{A}$	$\bar{B} \cap A$	$A \cap B$	$\bar{A} \cap B$	$\bar{B} \cap \bar{A}$	$\bar{B} \cap A$	$A \cap B$	$\bar{A} \cap B$	$\bar{B} \cap \bar{A}$	$\bar{B} \cap A$
„Bayesian text“	41%	32%	24%	24%	45%	39%	42%	33%	34%	31%	23%	26%	48%	45%	45%	45%
tree diagram	41%	38%	50%	59%	66%	75%	63%	75%	50%	53%	59%	56%	61%	58%	53%	58%
double tree	47%	44%	56%	53%	42%	45%	36%	42%	66%	63%	63%	66%	71%	74%	68%	71%
net diagram	77%	80%	77%	80%	76%	76%	76%	79%	75%	78%	78%	78%	74%	74%	65%	65%
2×2 table	94%	94%	100%	94%	88%	85%	85%	85%	64%	64%	64%	64%	71%	71%	71%	68%
